# The responses of rice plant to tricyclazole at the transcriptome and metabolome levels

**DOI:** 10.3389/fpls.2026.1723722

**Published:** 2026-02-03

**Authors:** Wengong Huang, Dongmei Shi, Aihua Cheng, Guofeng Chen, Feng Liu, Xiaobo Zhang, Jiannan Dong, Jing Lan, Hongbo Ren, Wei Guo, Baohai Liu

**Affiliations:** Safety and Quality Institute of Agricultural Products, Heilongjiang Academy of Agricultural Sciences, Harbin, Heilongjiang, China

**Keywords:** metabolome, rice, stress, transcriptome, tricyclazole

## Abstract

**Introduction:**

Tricyclazole is widely employed as a pesticide for controlling rice blast. While effective, it simultaneously acts as a stressor on rice plants.

**Methods:**

Rice seedlings were treated with tricyclazole for 7 days, and the root and shoot samples were collected for analysis. Four biomarkers of oxidative stress, including SOD, CAT, POD, and MDA were measured to evaluate the physiological responses. Integrated transcriptome and metabolome analysis was performed to reveal the mechanisms underlying the tricyclazole-induced stress response.

**Results:**

Tricyclazole significantly increased the levels of SOD, CAT, POD, and MDA in the root and shoot of rice. A variety of differential metabolites and differentially expressed genes (DEGs) were identified, which exhibited diverse functionalities. Flavonoid biosynthesis, glutathione metabolism, and phenylpropanoid biosynthesis were three important metabolic pathways in response to tricyclazole. These pathways encompassed many important metabolites and regulatory genes involved in stress responses. In addition, D-mannose, hispidulin-8-C-glucoside, dicumarol, and 4-hydroxyderricin in the root, and S-Adenosyl-L-methionine, nicotinate D-ribonucleoside, luteolin-7-O-(3'-O-coumaroyl) sophorotrioside, and D-Arabinono-1,4-lactone in the shoot were key metabolites involved in self-detoxification and resistance development of rice against tricyclazole. At the molecular level, these metabolites could be regulated by various DEGs involved in the systemic acquired resistance and response to toxic substances.

**Discussion:**

A coordinated molecular and metabolic reprogramming triggered by tricyclazole enables the rice plant to mitigate stress and develop resistance.

## Introduction

Rice (*Oryza sativa L.*), belongs to the genus Oryza within the family *Poaceae*, is one of the most important cereal crops (Kandil et al., 2022). Globally, rice ranks as the third most produced agricultural commodity and serves as a staple food for approximately half of the human population (Gross and Zhao, 2014; Nazish et al., 2022). In agricultural production, adversity stress is the most important factor affecting crop yield and quality. Rice is particularly susceptible to a range of abiotic stresses, such as drought, cold, salinity, and heavy metal toxicity, as well as biotic stresses caused by pathogens, such as fungi, bacteria, and viruses (Ahmad, 2022). Given that stress generates effects at the phenotypic, physiological, biochemical, and molecular levels, studying the mechanisms of stress response has become a key focus of agricultural research.

Rice blast is a widespread and destructive disease affecting cultivated rice globally, posing a serious threat to production and food security (Fernandez et al., 2021). Under favourable environmental conditions, the yield loss attributed to rice blast can reach 30–50% in major rice-growing regions (Ashkani et al., 2015). This devastating fungal disease is primarily caused by *Magnaporthe oryzae*, a hemibiotrophic pathogen that infects rice by initially maintaining host cells in the early phase, and subsequently kills cells in the late stage (Syauqi et al., 2022). During infection and colonisation, *Magnaporthe oryzae* mainly targets immune responses, destroys host defenses, and enhances the susceptibility of rice plants to fungal invasion (Fernandez and Orth, 2018). So far, pesticide application is one of the effective strategies to control rice blast, among which tricyclazole (8-methyl-[1,2,4]triazolo[3,4-b][1,3]benzothiazole) is one of the most common ones. As a triazole fungicide, tricyclazole is effective to control rice blast by inhibiting spore germination and adherent spore formation (Li et al., 2023; Liu et al., 2024c). However, tricyclazole, especially at high doses, also causes certain physiological toxicity to rice, thereby affecting rice growth and quality. Elucidating the underlying mechanisms of rice’s response to tricyclazole provides a theoretical basis for developing resistant varieties and formulating targeted strategies to reduce phytotoxicity, thereby supporting sustainable rice yield and quality.

Pesticides are a form of stress in plants, and their absorption in leaves or roots can lead to adverse outcomes, such as tissue damage, growth inhibition, and quality decline. In terms of underlying mechanisms, pesticides often trigger molecular-level changes and in turn, disrupt crucial metabolic processes (Liu et al., 2024a). Transcriptome and metabolome sequencing offer great possibilities for elucidating the intrinsic mechanisms of plants’ responses to pesticide stress, among which the former identifies key regulatory genes, while the latter captures dynamic changes in metabolic profiles (Lv et al., 2024). So far, some studies have confirmed relevant applications in rice. As reported, butachlor affects carbohydrate metabolism, and the key differentially expressed genes (DEGs) are involved in starch and sucrose metabolism (Liu and Zhu, 2020). Chlorpyrifos affects amino acid metabolism, and the key DEGs are involved in aspartate and glutamate metabolism (Liu and Zhu, 2020). Ametryn affects the metabolisms of carbohydrates, amino acids, hormones and phenylpropanoids in rice, and relevant genes encoding enzymes for the catabolism of ametryn are up-regulated (Qiao et al., 2023). ZhiNengCong elevates the levels of carbohydrates, vitamins, nucleotides, and amino acids in rice with bacterial leaf streak, and up-regulates genes involving resistance, growth, and sugar/amino acids/lipid metabolisms (Lu et al., 2025). In the present study, transcriptome and metabolome sequencing were performed in rice plants treated with tricyclazole. The metabolomic and transcriptomic changes were analysed in the root and shoot samples. This study aims to elucidate the mechanisms of stress defense and resistance establishment in response to tricyclazole, and to identify pivotal metabolites and regulatory genes involved.

## Materials and methods

### Sample processing

Rice (Wuyou 4) was used for the hydroponic cultivation of seedlings. When the seedlings reached the stage of one-leaf and one-heart (about 10 days after germination), the medium containing 20 mg/L tricyclazole was refreshed to trigger a stress response (P group). The seedlings cultured in conventional medium without tricyclazole were used as an internal check (CK group). The CK and P groups both have three biological replicates, with 50 seedlings per replicate. After 7 days of continuous cultivation, the seedlings were collected and then divided into the root and shoot parts. Four biomarkers of oxidative stress, including SOD, CAT, POD, and MDA were measured in the root and shoot samples as previously described (Chen et al., 2025). For the above physiological assay, three independent experiments were conducted, with three biological replicates in each group. Another part of the root and shoot samples (a total of 4 groups; N = 3 per group) was used for transcriptome and metabolome sequencing as follows.

### Transcriptome sequencing

RNAs were isolated from samples of different groups using CTAB (Sangon, Shanghai, China) combined with pBIOZOL (Bioer, Hangzhou, China), quantified by a fluorescence analyser (Qubit 4.0, Thermo Fisher Scientific, Waltham, MA, USA), and assessed by a high-throughput biological fragment analyser (Qsep400, Bioptic, Taiwan, China). Based on qualified RNAs, libraries were constructed using an RNA-seq library prep kit (Vazyme, Nanjing, China), quantified by a fluorescence analyser (Qubit 4.0), and assessed by a fragment analyser (Agilent 2100, Santa Clara, CA, USA). Finally, a DNA nanoball (about 300 copies) was prepared from a qualified library and sequenced on the MGI platform (BGI Genomics, Shenzhen, China).

### Transcriptome analysis

Raw data were processed using fastp v0.23.2, and the reads with adapters, with N > 10% of the base number of the reads, and with Q ≤ 25 (low quality) for more than 50% of the base number of the reads were filtered out. The number of raw reads, clean reads, Q20, Q30, and GC content of each sample were calculated. Clean reads were mapped to the reference genome (IRGSP-1.0_genome) using HISAT2 v2.2.1. Fragments Per Kilobase of transcript per Million fragments mapped (FPKM) was calculated to reflect gene expression levels using featureCounts v2.0.3. Principal component analysis (PCA) was performed using prcomp (R v4.1.2) to reveal the inter-group and intra-group variabilities. For two-group comparison, DEGs were determined using DESeq2 v1.22.1, and the threshold was log2FC ≥ 1 and False Discovery Rate (FDR) < 0.05 (Benjamini-Hochberg test). Based on the isolated DEGs, clustered heatmaps and volcano plots were generated. Besides, DEGs were aligned with KOG databases using diamond v2.0.9 to obtain annotation results.

### Metabolome sequencing

The samples were frozen in a lyophiliser (Scientz-100F, Scientz, Ningbo, China) and ground into powder using a grinder (MM 400, Retsch, Haan, Germany). Subsequently, 30 mg powder was added into 1500 μL pre-cooled 70% methanolic aqueous internal standard extract. The mixture was vortexed for 30 s with 30 min intervals for six times in total. After 3 min of centrifugation at 12,000 rpm, the supernatant was collected and filtered through a microporous membrane (0.22 μm). The filtrate was used for metabolome sequencing using an UPLC-ESI-MS/MS system (SCIEX, Shanghai, China).

### Metabolome analysis

The MS data were qualitatively analysed based on the MWDB database (Myway, Shenzhen, China). The isotope signals, repeated signals containing K^+^, Na^+^, NH^4+^, and repeated signals themselves are fragment ions of other substances with higher molecular weight were removed during analysis. In addition, the metabolites were quantified using the multiple reaction monitoring mode. PCA was performed using prcomp (R v4.1.2) to reveal the inter-group and intra-group variabilities. For two-group comparison, differential metabolites were determined by VIP > 1, fold change ≥ 2/≤ 0.5, and P < 0.05. VIP values were generated from the OPLS-DA (Orthogonal Partial Least Squares-Discriminant Analysis) model using MetaboAnalystR. A permutation test (200 permutations) was performed to avoid overfitting (**Supplementary Figure S1**). Based on the isolated differential metabolites, clustered heatmaps, volcano plots, and scatter plot were generated. Besides, Z-score standardization was used to reveal the distribution of important differential metabolites between groups.

### Integrated analysis of metabolome and transcriptome

Based on the DEGs and differential metabolites, the KEGG pathways annotated by both omics were identified. If both the metabolomic and transcriptomic data have enrichment results in MetMap pathway (Myway), the co-annotated MetMap was added to the results of KEGG. If no MetMap pathways were co-annotated, only the KEGG results were displayed. Besides, DEGs and differential metabolites with |Pearson correlation coefficient| > 0.8 and P < 0.05 were used to draw cornetwork plots using Cytoscape v3.8.

### Targeted validation of key metabolites

D-mannose and S-adenosyl-L-methionine were selected for targeted validation based on the commercial availability of reliable reference standards and the existence of well-established quantitative detection methods.

The concentration of D-mannose in the root was measured by gas chromatography-mass spectrometry (GC-MS). Simply, freeze-dried samples were ground and 20 mg powder samples were immersed in 500 µL methanol:isopropanol:water (3:3:2, v/v/v). After vortexing, sonication, and centrifugation, 50 µL supernatants were mixed with 20 μL internal standards. For accharide analysis, the internal standards were [²H_6_]-D-ribose (IR-25184; isoreag, Shanghai, China) and adonitol (R017943, Shanghai, China), prepared as a 1000 µg/mL solution in methanol. After dried under a nitrogen stream, the residue was derivatized by reaction with 100 μL methoxyamine hydrochloride in pyridine at 37 °C for 2 h, followed by reaction with 100 μL BSTFA at 37 °C for 30 min. Finally, 50 μL derivatization solution was diluted to 1 mL with n-hexane, filtered through a 0.22 μm membrane, and used for GC-MS analysis on the Agilent 8890-5977B platform (Agilent, Santa Clara, CA, USA). Based on the quantitative ion (m/z, 160) and qualitative ion (m/z, 319), a selected ion monitoring detection mode was employed to ensure the resolution of the target compound. The retention time (~7.54 min) and ion ratio (~102) of D-mannitol in the test samples matched those in the mixed standards, confirming accurate identification. For quality control, three quality control samples (pooled samples) were inserted between the mixed standards and the test samples during loading. The method performance characteristics were as follows: inter-day precision (n = 3), 9.34%; intra-day precision (n = 3), 9.10%; recovery, 93.07%. The calibration curve was y = 0.535674x – 2.162182E-004.

The concentration of S-adenosyl-L-methionine in the shoot was measured by liquid chromatography-mass spectrometry (LC-MS). Simply, 50 mg thawed samples were extracted in 500 μL methanol containing 10 μL internal standard for 30 min at -20 °C. The internal standard was [^2^H_3_]-SAM (SAM-_d3_) (485-80-3; SCRSTANDARD, Shanghai, China), prepared as a 500 ng/mL solution in methanol. After centrifugation, 250 μL supernatant was collected for LC-MS analysis on QTRAP 6500+ System (Sciex, Framingham, MA, USA). The ion mode was [M+H]+. Quantitative analysis was performed using the multiple reaction monitoring mode. The retention time of S-adenosyl-L-methionine (~5.68 min) in the test samples matched that in the mixed standards. For quality control, three quality control samples (pooled samples) were inserted between the mixed standards and the test samples during loading. The method performance characteristics were as follows: inter-day precision (N = 3), 6.36%; intra-day precision (N = 3), 5.87%; recovery, 128.80%. The calibration curve was y = 0.00943x - 0.03064.

### Statistical analyses

Physiological and verification experiments were performed with three independent biological replicates (N = 3 per group). Data were expressed as mean ± standard deviation. Statistical analysis was conducted using GraphPad Prism 7.0 (GraphPad, San Diego, CA, USA). Differences between two groups were assessed by independent sample t-test. A p-value < 0.05 was considered statistically significant.

## Results

### The physiological responses of rice plant to the treatment of tricyclazole

**Figure 1:**
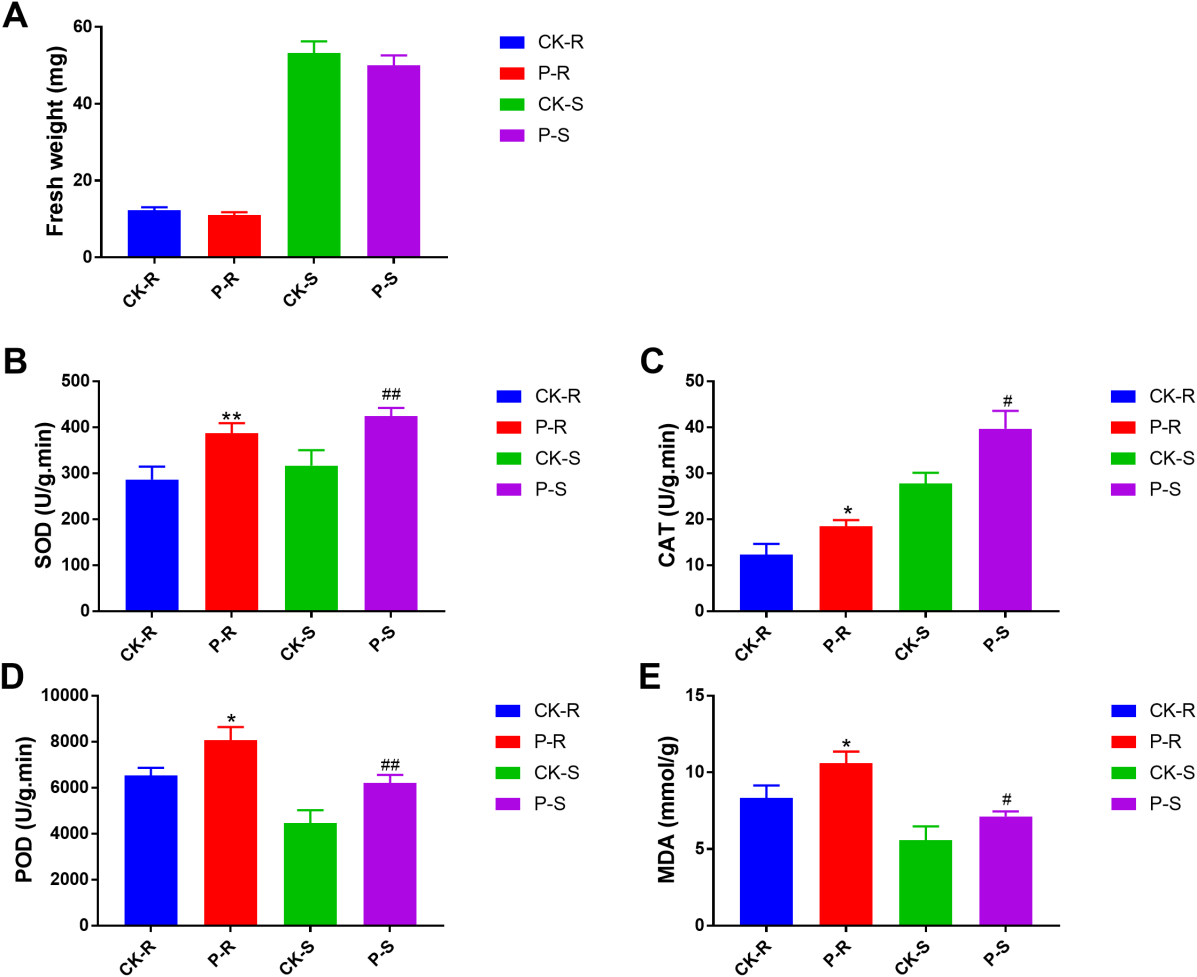
Physiological changes in the root and shoot of rice plant after the treatment of tricyclazole. **(A)** Fresh weight. **(B)** SOD. **(C)** CAT. **(D)** POD. **(E)** MDA. ^*^P < 0.05, ^**^P < 0.01 *vs.* CK-R; ^#^P < 0.05, ^##^P < 0.01 *vs.* CK-S.

After the treatment of tricyclazole, the physiological changes in rice plant were evaluated. As shown in **Figure 1A**, the fresh weights of the root and shoot were both slightly reduced by the treatment of tricyclazole, but the differences were not significant. Subsequently, the oxidative stress was analysed based on four biomarkers. In the root, the levels of SOD, CAT, POD, and MDA were significantly higher in the P-R group than in the CK-R group (P < 0.05). Consistently, the levels of SOD, CAT, POD, and MDA in the shoot were also significantly higher in the P-S group than in the CK-S group (P < 0.05) (**Figures 1B-E**).

### Changes of transcriptomes in the root and shoot of rice plant after the treatment of tricyclazole

**Figure 2:**
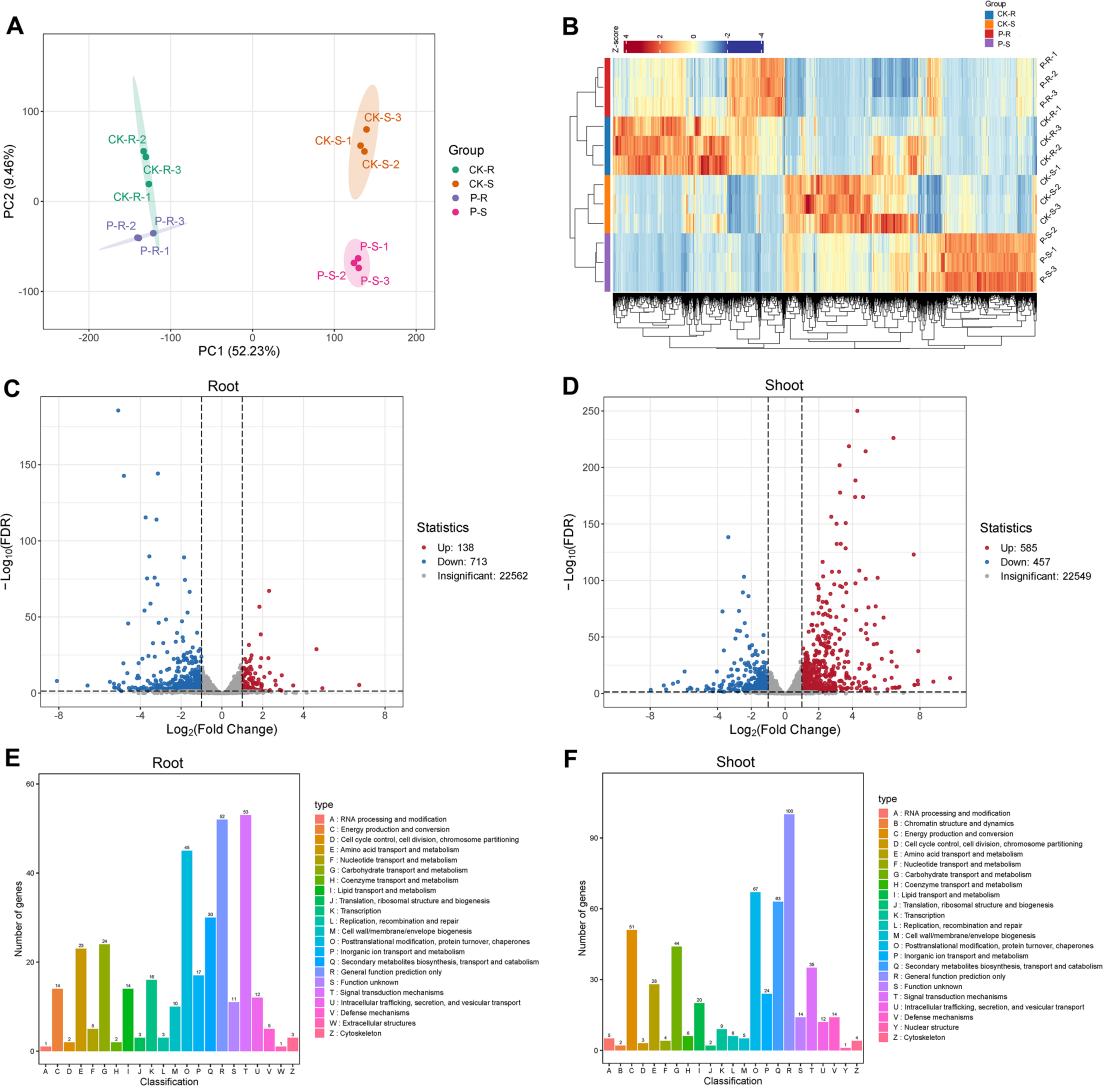
Transcriptomic differences in the root and shoot of rice plant after the treatment of tricyclazole. **(A)** PCA; different colored dots represent samples from different groups. **(B)** Cluster heatmap; red represents up-regulation and blue represents down-regulation. **(C)** Volcanic diagram in the root. **(D)** Volcanic diagram in the shoot; green points represent down-regulated DEGs, red points represent up-regulated DEGs, and gray points represent genes without significant differences. **(E)** Column diagram of KOG annotation in the root. **(F)** Column diagram of KOG annotation in the shoot.

High-throughput sequencing was performed to reveal the transcriptomic differences in rice plant after the treatment of tricyclazole. Both the RNA samples and the sequencing data are of high quality (**Supplementary Table S1** and **2**). PCA displayed a high intra-group repeatability, evidenced by the fact that all intra-group replicates were clustered within a confidence ellipse. In addition to a slight overlap between the CK-R and P-R (treatment) groups, the four groups exhibited separated confidence ellipses, which indicated potential differences at the transcription level (**Figure 2A**). Cluster heatmap showed the presence of a large number of DEGs between the CK and P groups in the root and shoot samples (**Figure 2B**). According to the criteria of log2FC ≥ 1 and FDR < 0.05, 138 up-regulated and 713 down-regulated DEGs were determined in the root, and 585 up-regulated and 457 down-regulated DEGs were determined in the shoot between the CK and P groups (**Figures 2C, D**). KOG annotation revealed a diverse range of functional roles in these DEGs, with distinct profiles observed between the root and shoot (**Figures 2E, F**).

### Changes of metabolomes in the root and shoot of rice plant after the treatment of tricyclazole

**Figure 3:**
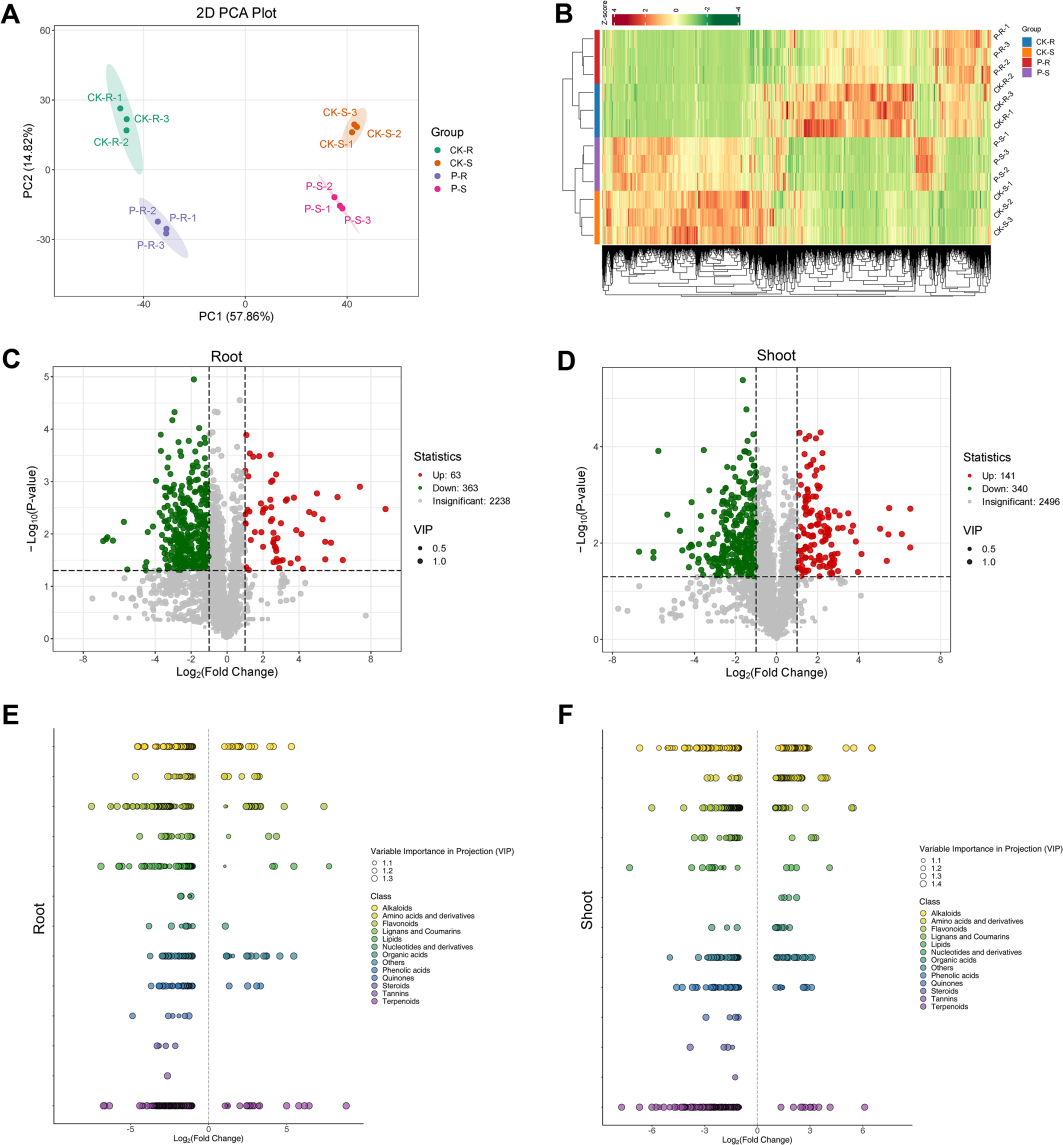
Metabolomic differences in the root and shoot of rice plant after the treatment of tricyclazole. **(A)** PCA; different colored dots represent samples from different groups. **(B)** Cluster heatmap; red represents up-regulation and green represents down-regulation. **(C)** Volcanic diagram in the root. **(D)** Volcanic diagram in the shoot; green points represent down-regulated metabolites, red points represent up-regulated metabolites, and gray points represent metabolites without significant differences. **(E)** Scatter plot in the root. **(F)** Scatter plot in the shoot; each point represents a metabolite, and different colours indicate different classifications.

UPLC-MS/MS was further performed to reveal the metabolomic differences in rice plant after the treatment of tricyclazole. A total of 3058 metabolites were detected before filtering. PCA showed that the intra-group replicates were all within a confidence ellipse, indicating a good repeatability. A clear separation among the four groups indicates the potential differences at the metabolic level (**Figure 3A**). Between the CK and P groups in the root and shoot, a large number of differential metabolites were observed on the cluster heatmap (**Figure 3B**). According to the criteria of VIP > 1, fold change ≥ 2/≤ 0.5, and P < 0.05, 63 up-regulated and 363 down-regulated metabolites were determined in the root, and 141 up-regulated and 340 down-regulated metabolites were determined in the shoot between the CK and P groups (**Figures 3C, D**). These differential metabolites have a wide range of categories, and some metabolites within the same category may also have differences between the root and shoot (**Figures 3E, F**).

### Functional enrichment of differential metabolites and DEGs

**Figure 4:**
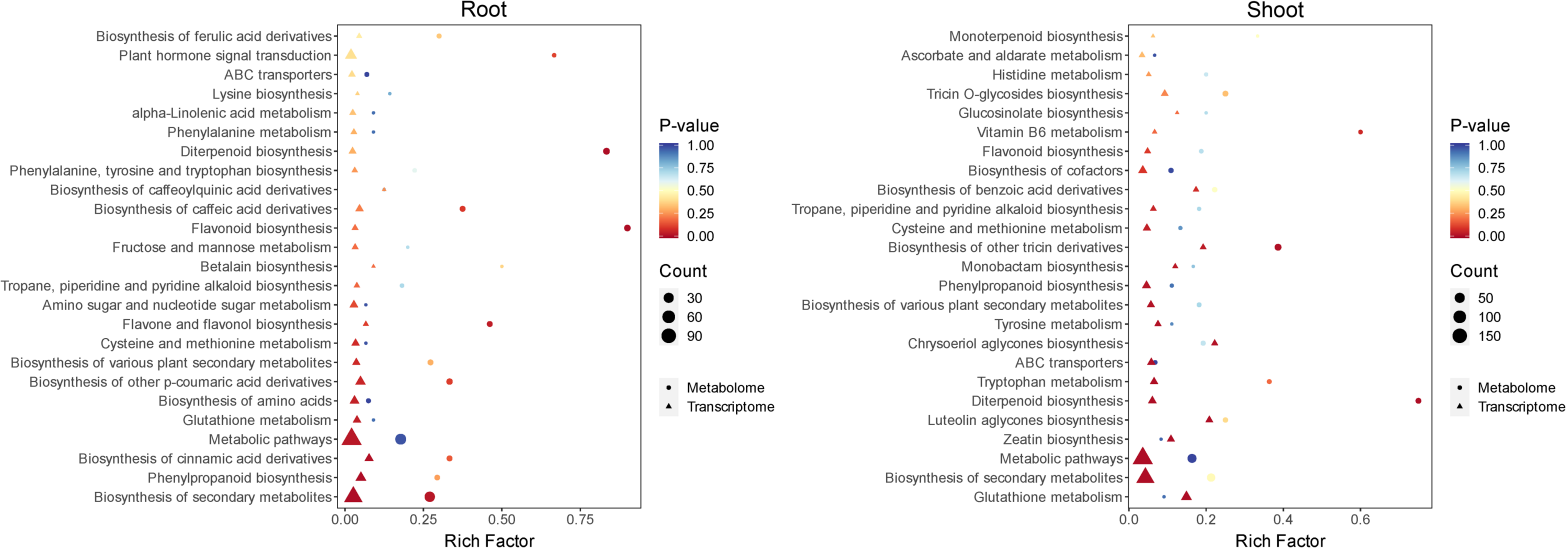
Bubble plots of co-enriched KEGG by differential metabolites and DEGs in the root and shoot. Red, yellow, and blue represent a change in the significance of enrichment from high, medium, to low; the shape of bubbles represents different omics, and the size of bubbles represents the number of differential metabolites and DEGs.

Bubble plots were constructed using KEGG pathways co-enriched by two omics datasets. The top 25 terms were listed in **Figure 4**. In the root, the differential metabolites and DEGs were mainly co-enriched in the flavonoid biosynthesis, flavone and flavonol biosynthesis, phenylpropanoid biosynthesis, glutathione metabolism, biosynthesis of secondary metabolites, biosynthesis of amino acids, etc. In addition to the flavonoid biosynthesis, glutathione metabolism, phenylpropanoid biosynthesis, and biosynthesis of secondary metabolites, the differential metabolites and DEGs in the shoot were also co-enriched in zeatin biosynthesis, luteolin aglycones biosynthesis, diterpenoid biosynthesis, tryptophan metabolism, chrysoeriol aglycones biosynthesis, etc.

### Key metabolic pathways in response to tricyclazole

**Figure 5:**
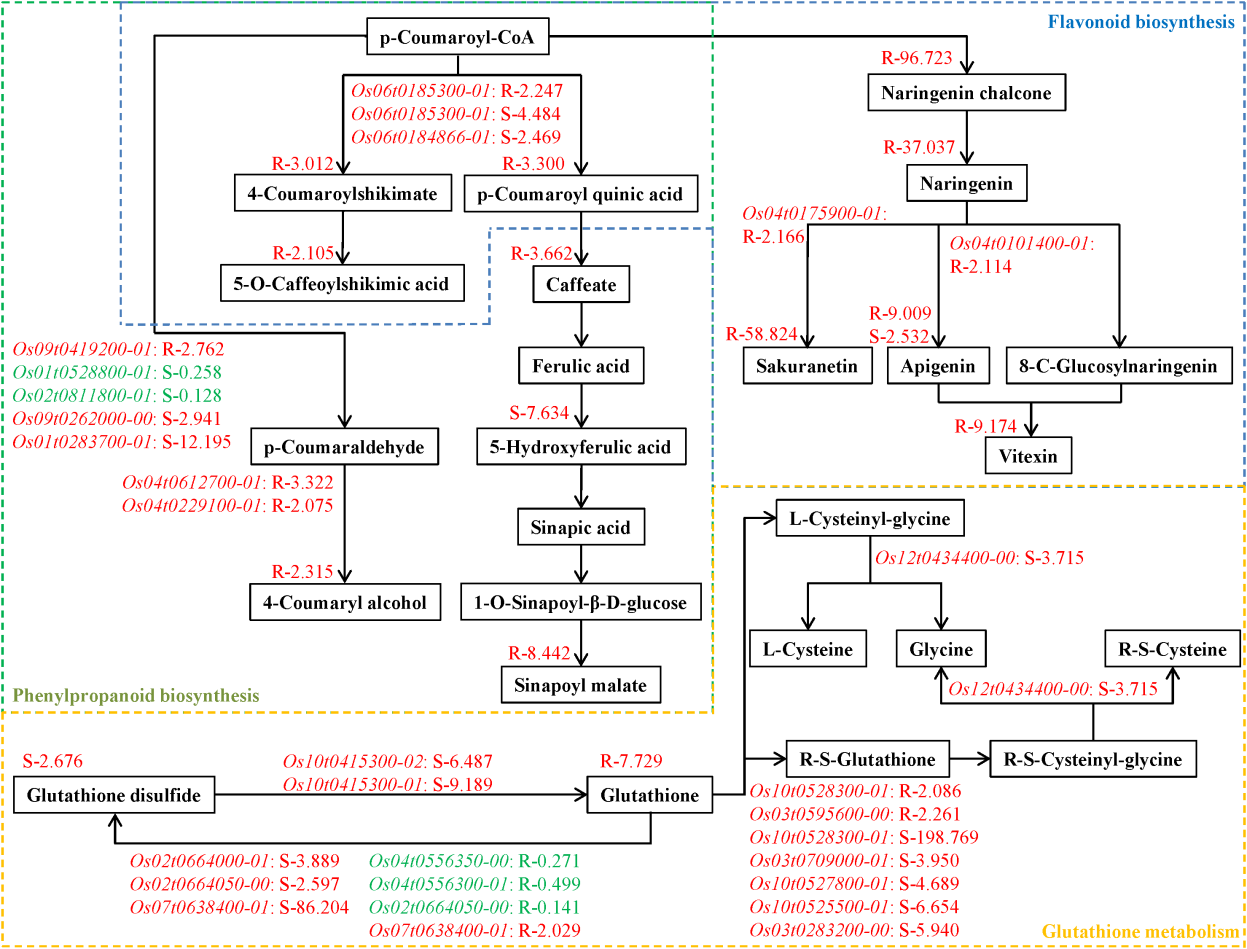
A schematic diagram depicting key metabolic pathways, including flavonoid biosynthesis, glutathione metabolism, and phenylpropanoid biosynthesis. Genes and metabolites are colored red or green to indicate up- or down-regulation, based on their fold changes in the root and shoot.

Based on the key metabolic pathways (flavonoid biosynthesis, glutathione metabolism, phenylpropanoid biosynthesis) enriched in both the root and shoot samples, a metabolic diagram was constructed to display relevant metabolites and regulatory genes (**Figure 5**). In flavonoid biosynthesis, tricyclazole increased sakuranetin and apigenin levels in the root, which might be mediated by *Os04t0175900–01* and *Os04t0101400-01*, respectively. In phenylpropanoid biosynthesis, tricyclazole induced the accumulation of 4-Coumaryl alcohol in the root, possibly mediated by *Os04t0612700–01* and *Os04t0229100-01*. In addition, 4-Coumaroylshikimate and p-Coumaroyl quinic acid were differential metabolites involved in both the flavonoid biosynthesis and phenylpropanoid biosynthesis, and their accumulation in the root might be positively regulated by *Os06t0185300-01.* Besides, glutathione disulfide is a key metabolite in glutathione metabolism. Under tricyclazole treatment, the up-regulation of *Os02t0664000-01*, *Os02t0664050-00*, and *Os07t0638400–01* might contribute to the accumulation of glutathione disulfide in the shoot.

### Identification of key metabolites and relevant genes involved in stress response

**Figure 6:**
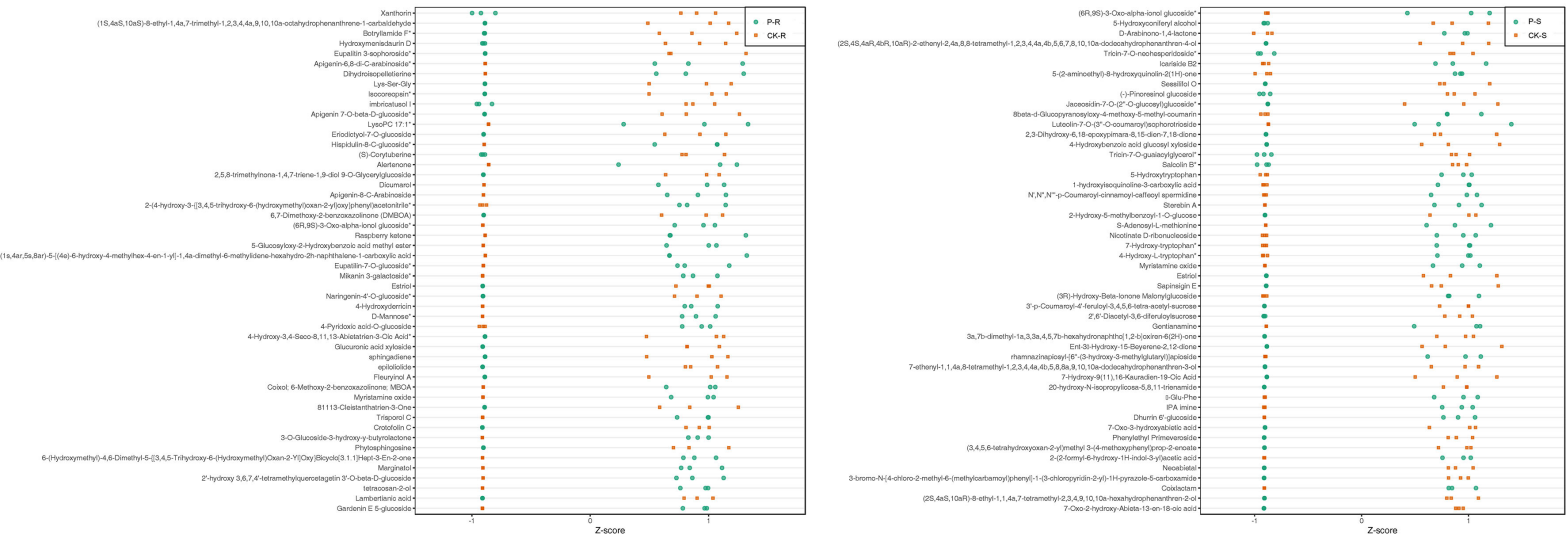
The Z-value plots of differential metabolites (top 50) in the root and shoot. Green and yellow dots represent the values of metabolites in the P and CK groups, respectively.

**Figure 7:**
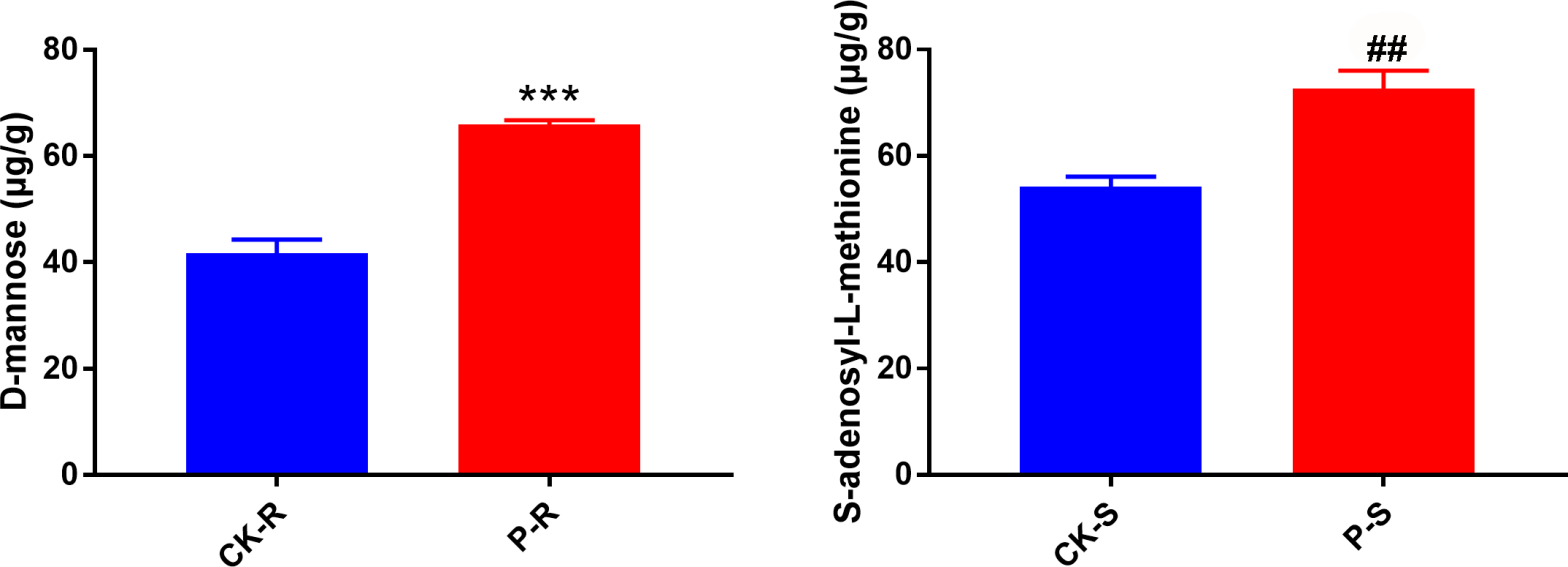
Validation of D-mannose and S-adenosyl-L-methionine concentrations by targeted GC -MS and LC-MS, respectively. ^***^P < 0.001 *vs.* CK-R; ^##^P < 0.01 *vs.* CK-S.

**Figure 8:**
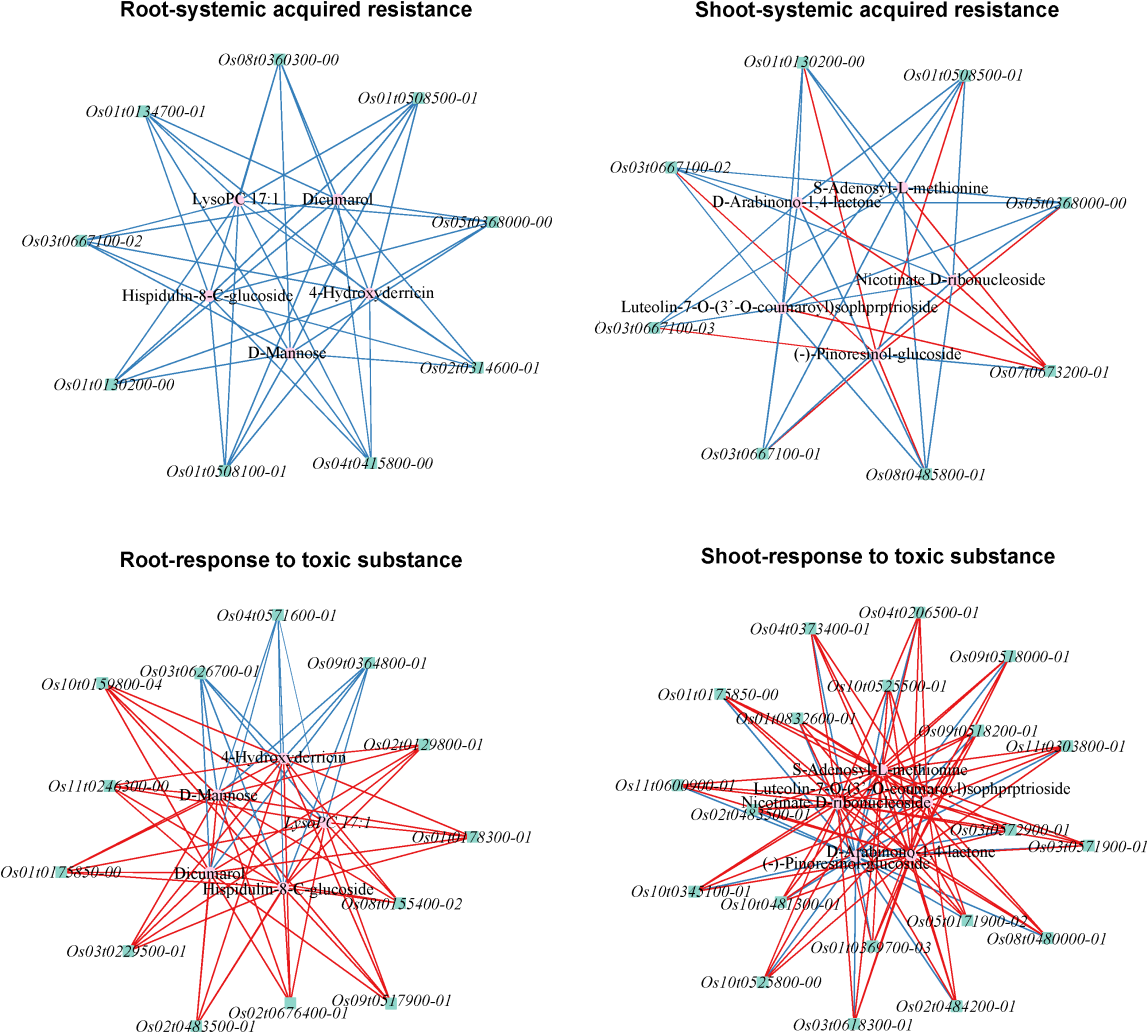
Correlation network plots constructed by DEGs implicated in the systemic acquired resistance and the response to toxic substances, and the screened metabolites.

Referring to Z-score, the top 50 differential metabolites in the root and shoot were displayed in **Figure 6**. These metabolites may be important participants in rice’s response to tricyclazole. For example, D-mannose, hispidulin-8-C-glucoside, dicumarol, and 4-hydroxyderricin were all increased by the treatment of tricyclazole in the root. In the shoot, tricyclazole treatment increased S-Adenosyl-L-methionine, nicotinate D-ribonucleoside, luteolin-7-O-(3’-O-coumaroyl)sophorotrioside, and D-Arabinono-1,4-lactone. Targeted GC-MS subsequently validated that the concentration of D-mannose in the root was significantly higher in the P-R group than in the CK-R group (65.89 ± 0.52 *vs.* 41.72 ± 1.49 μg/g, P < 0.001). Targeted LC-MS validated that the concentration of S-Adenosyl-L-methionine in the shoot was significantly higher in the P-S group than in the CK-S group (72.64 ± 1.97 *vs.* 54.20 ± 1.08 μg/g, P < 0.01) (**Figure 7**). Potential molecular networks for these metabolites were further constructed. As shown in **Figures 8**, nine and eight DEGs implicated in the systemic acquired resistance were revealed in the root and shoot, respectively. Additionally, 13 and 20 DEGs implicated in the response to toxic substances were revealed in the root and shoot samples, respectively. These DEGs may be involved in the response to tricyclazole by mediating stress response and resistance development.

## Discussion

Tricyclazole is a pesticide widely used for the treatment of rice blast (Li et al., 2023; Liu et al., 2024c). Although tricyclazole effectively inhibits *Magnaporthe oryzae* infection, it simultaneously triggers defense responses in rice, imposing a form of stress on the plant. In the present study, the treatment of tricyclazole significantly increased the levels of SOD, CAT, POD, and MDA in the root and shoot of rice plants. These results indicate that tricyclazole induces a state of oxidative stress in rice plants, which in turn activates a systemic antioxidant defense response. Subsequently, metabolome and transcriptome sequencing were performed to reveal the mechanisms underlying the responses of rice plant to tricyclazole. As a result, a variety of differential metabolites and DEGs were isolated from the root and shoot samples. These factors may be key responders to tricyclazole and participate in a coordinated defense response in rice plants.

In order to determine which metabolic pathways are affected by tricyclazole, co-enrichment analysis was performed on the differential metabolites and DEGs. Integrated analysis showed that the flavonoid biosynthesis, glutathione metabolism, and phenylpropanoid biosynthesis were key metabolic pathways enriched in both the root and shoot samples. As reported, phenylpropanoids are a group of secondary metabolites that are associated with tolerances to drought stress (Shim et al., 2025), soluble aluminum (Wang et al., 2023), and ultraviolet B radiation (Liu et al., 2024b) in rice. As a branch of phenylpropanoid, flavonoids are also important in response to abiotic and biotic stresses in plants (Ahmed et al., 2021; Chen et al., 2023). In rice, flavonoids help to combat drought and ultraviolet radiation stress (Jan et al., 2022), salt and heat stress (Jan et al., 2021), and thiocyanate pollution (Tian et al., 2024). Similarly, glutathione is also an essential metabolite in plants that mediates the responses to both abiotic and biotic stresses by virtue of its roles in detoxification, antioxidant defense, and redox homeostasis (Noctor et al., 2012). Evidence has determined that glutathione helps rice resist complete submergence, chromium toxicity, and salt stress. The above evidence supports that these metabolic pathways may play a central role in rice’s response to tricyclazole. Furthermore, to better elucidate the involved metabolites and genes, a metabolic diagram was constructed. This diagram highlights several important metabolites and regulatory genes potentially implicated in tricyclazole stress response. For example, naringenin 7-O-methyltransferase encoded by *Os04t0175900–01* increases the accumulation of sakuranetin, a crucial defense metabolite against environmental stress (Miyamoto, 2025). *Os04t0101400-01*, which encodes a flavone synthase II, is involved in the synthesis of apigenin, contributing to antioxidant activity and stress tolerance in plants (Zhu et al., 2024). *Os04t0612700–01* and *Os04t0229100–01* both encode cinnamyl-alcohol dehydrogenase, a key enzyme involved in stress defense in plants, are associated with the accumulation of 4-Coumaryl alcohol (Park et al., 2018; Bagniewska-Zadworna et al., 2014). In addition, glutathione peroxidase encoded by *Os02t0664000-01*, *Os02t0664050-00*, and *Os07t0638400–01* can eliminate peroxides by oxidizing reduced glutathione to glutathione disulfide (Averill-Bates, 2023). To sum up, tricyclazole may activate the stress response of rice plants by affecting the flavonoid, glutathione, and phenylpropanoid metabolic pathways at both the transcriptome and metabolome levels.

Further screening was conducted on potential metabolites involved in stress response. Some metabolites may be critical in responding to tricyclazole-induced stress. For example, Hispidulin 8-C-(2- Osyringoyl-b-glucopyranoside) is involved in antioxidant defence of *Haberlea rhodopensis* to cold acclimation and relevant desiccation (Georgieva et al., 2021). Ascorbic acid is an important participant in scavenging ROS and blocking photoinhibition in plants under stress (Celi et al., 2023; Yang et al., 2023a). Nicotinate D-ribonucleoside, the ribonucleoside form of nicotinic acid, serves as a key intermediate in the salvage pathway of nicotinamide adenine dinucleotide (NAD) biosynthesis in plants. NAD-mediated ROS plays a significant role in controlling plant growth and conferring stress resistance (Hong et al., 2020). Elevated levels of nicotinate D-ribonucleoside have been observed in Cu(II)-exposed yeast (Farrés et al., 2016), microbial inoculated rhizospheric soil (Lai et al., 2024), and downy mildew-infected sunflower (Zhu et al., 2023). Luteolin-7-O-(3’-O-coumaroyl) isophorotrioside may also be implicated in stress responses by acting as a derivative of luteolin. Luteolin is involved in plants’ resistance to *Sclerotinia sclerotiorum* (Robison et al., 2018), *Mythimna separata* (Qi et al., 2016), nitrogen deficiency (Kováčik and Klejdus, 2014), and Cd (Zhu et al., 2024). D-Arabinono-1,4-lactone serves as a direct precursor in the biosynthetic pathway of L-ascorbic acid (AsA). As one of the most pivotal antioxidants in plants, AsA contributes to plant resistance against a range of abiotic stresses (Wu et al., 2024b). Bao et al. reported that D-Arabinono-1,4-lactone enhances the tolerances to drought and chilling in tobacco and stylo (Bao et al., 2016). In the present study, consistent changes in the above metabolites were observed by metabolomics analysis in tricyclazole-treated rice, either in the root or in the shoot. The changes in these metabolites reflect a metabolic reprogramming, likely contributing to the activation of defence mechanisms. Besides, dicumarol and 4-hydroxyderricin are associated with antibacterial activity. 4-Hydroxyderricin is a chalcone that exhibits an antimicrobial activity against various pathogens, including viruses, bacteria, fungi, and protozoa (Caesar et al., 2018; Adhikari et al., 2025). Beyond its antimicrobial property, dicumarol also exhibits insecticidal activity and the ability to induce phytoalexin production in plants (Li et al., 2014; Rehman et al., 2017). These two metabolites may contribute to the antifungal property of tricyclazole against *Magnaporthe oryzae* in rice plants. It should be noted that the functional inferences for the above metabolites are inferred from studies in other plant species or under other stress conditions. Although these comparisons provide reasonable hypotheses for our findings, the specific physiological functions of these metabolites in tricyclazole-treated rice remain putative and require further validation through targeted studies.

D-mannose and S-adenosyl-L-methionine are also two important metabolites potentially involved in the response to tricyclazole. Consistent with the metabolomics results, verification experiments further confirmed the accumulation of D-mannose in the root and S-adenosyl-L-methionine in the shoot following tricyclazole treatment. D-mannose is a monosaccharide that elevated under salt stress in tomato (Wu et al., 2024a) and under cold stress in *Dendrobium huoshanense* (Wu et al., 2023). The role of D-Mannose in plant stress tolerance may be closely associated with ascorbic acid biosynthesis. As a key precursor, D-mannose is utilized in the D-mannose/L-galactose pathway to synthesize ascorbic acid (Mishra et al., 2024). The resulting ascorbic acid can scavenge ROS and enhance plant stress tolerance by regulating the antioxidant pool (Duan et al., 2016; Chen et al., 2020). The accumulation of D-mannose under tricyclazole treatment suggests the activation of an antioxidant defense mechanism centered on ascorbic acid biosynthesis. On the other hand, S-Adenosyl-L-methionine serves as a methyl donor in methylation reactions, which participates in the stress responses to *Ralstonia solanacearum* and alkali (Gong et al., 2014; Wang et al., 2019), drought (Peleg et al., 2011), freezing (Kou et al., 2018), and salt (Ma et al., 2017). Mechanistically, the involvement of S-adenosyl-L-methionine in stress responses is primarily linked to the ethylene, polyamine, and glutathione biosynthetic pathways, all of which are activated in plants under stress conditions (Yang et al., 2023b; Noctor et al., 2012). Specifically, 1-aminocyclopropane-1-carboxylic acid synthase catalyzes the cleavage of S-Adenosyl-L-methionine to produce 1-aminocyclopropane-1-carboxylic acid, which is then converted into ethylene by 1-aminocyclopropane-1-carboxylic acid oxidase. Ethylene can coordinate plant growth and stress management by interacting with auxin, abscisic acid, salicylic acid, jasmonic acid, and gibberellins (Shukla, 2025). After decarboxylation, S-adenosyl-L-methionine also acts as an aminopropyl donor in polyamine synthesis. Under stress, polyamines positively influence plant growth, ion balance, water retention, photosynthesis, and ROS production (Napieraj et al., 2023). As discussed earlier, glutathione metabolism is a key metabolic pathway in response to tricyclazole. In transsulfuration, the sulfur atom from S-adenosyl-L-methionine can be converted into cysteine through a series of enzymatic steps, making it a precursor for glutathione (Lu, 2000). Thus, the accumulation of S-adenosyl-L-methionine may enhance tricyclazole tolerance by activating the ethylene, polyamine, and glutathione biosynthesis.

Moreover, the molecular basis of the above differential metabolites was further explored by constructing correlation networks. The results showed that these metabolites were linked to a variety of DEGs implicated in the systemic acquired resistance and response to toxic substances. For example, NPR1 is an activator of systemic acquired resistance involved in plant immunity (Zavaliev et al., 2020). NPR1 plays a crucial role in enhancing plant resistance to biotic and abiotic stresses by modulating the interaction between salicylic acid and other defense and growth hormones (Zavaliev and Dong, 2023). Salicylic acid glucosyltransferase also acts an important regulator in plant defence response through its regulatory role in salicylic acid (Kobayashi et al., 2020). In the molecular networks, *Os03t0667100-02*, which encodes NPR1, was correlated with D-mannose and S-adenosyl-L-methionine. *Os09t0517900–01* and *Os04t0206500-01*, both encoding pathogen-inducible salicylic acid glucosyltransferase, were linked to D-mannose and S-adenosyl-L-methionine, respectively. These connections may be direct, or indirectly bridged via salicylic acid, with GDP-D-mannose/ascorbic acid and ethylene as key linkers (Miao et al., 2024; Mortimer et al., 2013; Shukla, 2025). Additionally, some other DEGs in the networks may also be key regulators in stress responses, such as *Os02t0314600-01* (aspartyl protease family protein) and *Os02t0483500-01* (BAHD acyltransferase), which correlate with D-mannose, as well as *Os07t0673200-01* (E3 ubiquitin-protein ligase BAH), *Os01t0175850-00* (UDP-glucosyltransferase), and *Os01t0369700-03* (glutathione S-transferase), which are linked to S-adenosyl-L-methionine. These defense-related genes may act through modulation of metabolic networks to ultimately coordinate the plant’s defense response.

**Figure 9:**
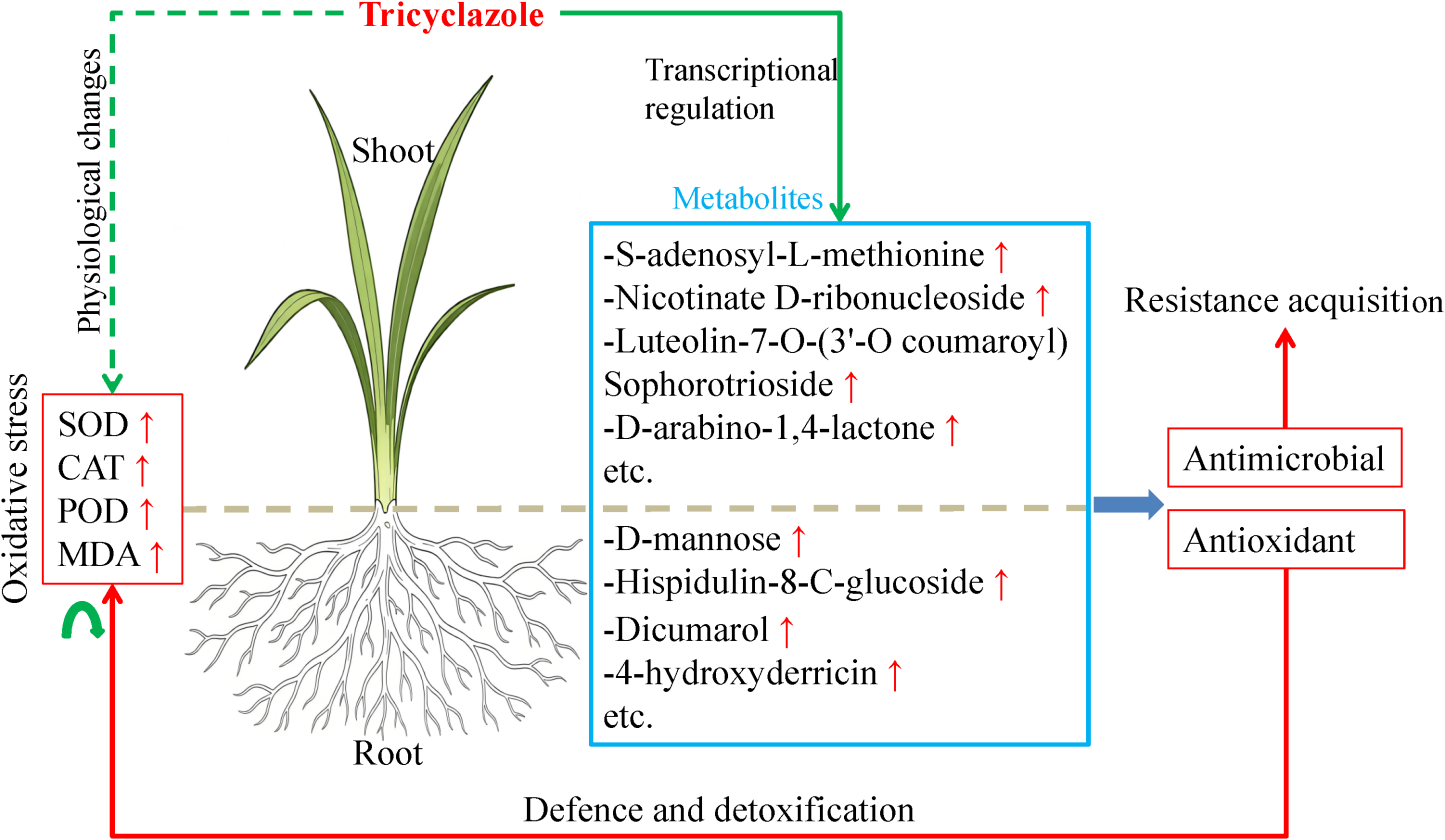
A schematic diagram of rice response to tricyclazole.

The possible mechanisms by which tricyclazole induces defence responses in rice plant are as follows. The root, acting as the direct site of tricyclazole perception, rapidly initiates transcriptional reprogramming. This reprogramming triggers a series of metabolic events, characterised by the accumulation of D-mannose, hispidulin-8-C-glucoside, dicumarol, 4-hydroxyderricin, etc. Among these, the first two metabolites exhibit antioxidant potential, and the latter two exhibit antimicrobial activity. Their enrichment suggests that the root system reinforces its chemical defence barrier to mitigate the potential adverse effects of tricyclazole and also to counteract potential secondary infections. As the response is transduced to the shoot via systemic signaling, it continues to trigger coordinated transcriptional reprogramming and associated metabolic adjustments. The accumulation of key metabolites, including S-adenosyl-L-methionine, nicotinate D-ribonucleoside, flavonoid luteolin-7-O-(3’-O-coumaroyl)sophorotrioside, and D-arabino-1,4-lactone, collectively illustrates the metabolic investment of the shoot system in antioxidant defence mechanisms. These metabolic adaptations may contribute to the adaptive detoxification of tricyclazole in rice plant, thereby helping to counteract the increase in oxidative stress (**Figure 9**).

This study remain has some limitations. First, the use of a single concentration and exposure time may not fully capture the dynamic effects of tricyclazole. Second, validation experiments relying on a single time point may miss critical early peaks or dynamic patterns and therefore limit mechanistic interpretation. Third, the proposed mechanisms remain preliminary, and future studies are needed to confirm the specific roles of key genes and metabolites identified.

## Conclusion

In conclusion, tricyclazole activated oxidative stress in the roots and shoots of rice plants. Flavonoid biosynthesis, glutathione metabolism, and phenylpropanoid biosynthesis were the main metabolic pathways in response to tricyclazole. D-mannose, hispidulin-8-C-glucoside, dicumarol, 4-hydroxyderricin (root), S-Adenosyl-L-methionine, nicotinate D-ribonucleoside, luteolin-7-O-(3’-O-coumaroyl) sophorotrioside, and D-Arabinono-1,4-lactone (shoot) might be key metabolites involved in self-detoxification and resistance development in tricyclazole-treated rice. In addition, transcriptomic reprogramming orchestrated the metabolic changes under tricyclazole treatment, and some associated DEGs were implicated in the systemic acquired resistance and response to toxic substances.

## Data Availability

The data presented in this study are deposited in the SRA repository, accession number: PRJNA1359757 (https://www.ncbi.nlm.nih.gov/bioproject/PRJNA1359757).
