# Supplementary figures and images for: The responses of rice plant to tricyclazole at the transcriptome and metabolome levels
Source: Front Plant Sci. 2026 Feb 3;17:1723722. doi: 10.3389/fpls.2026.1723722 (PMC12909226; doi:10.3389/fpls.2026.1723722)

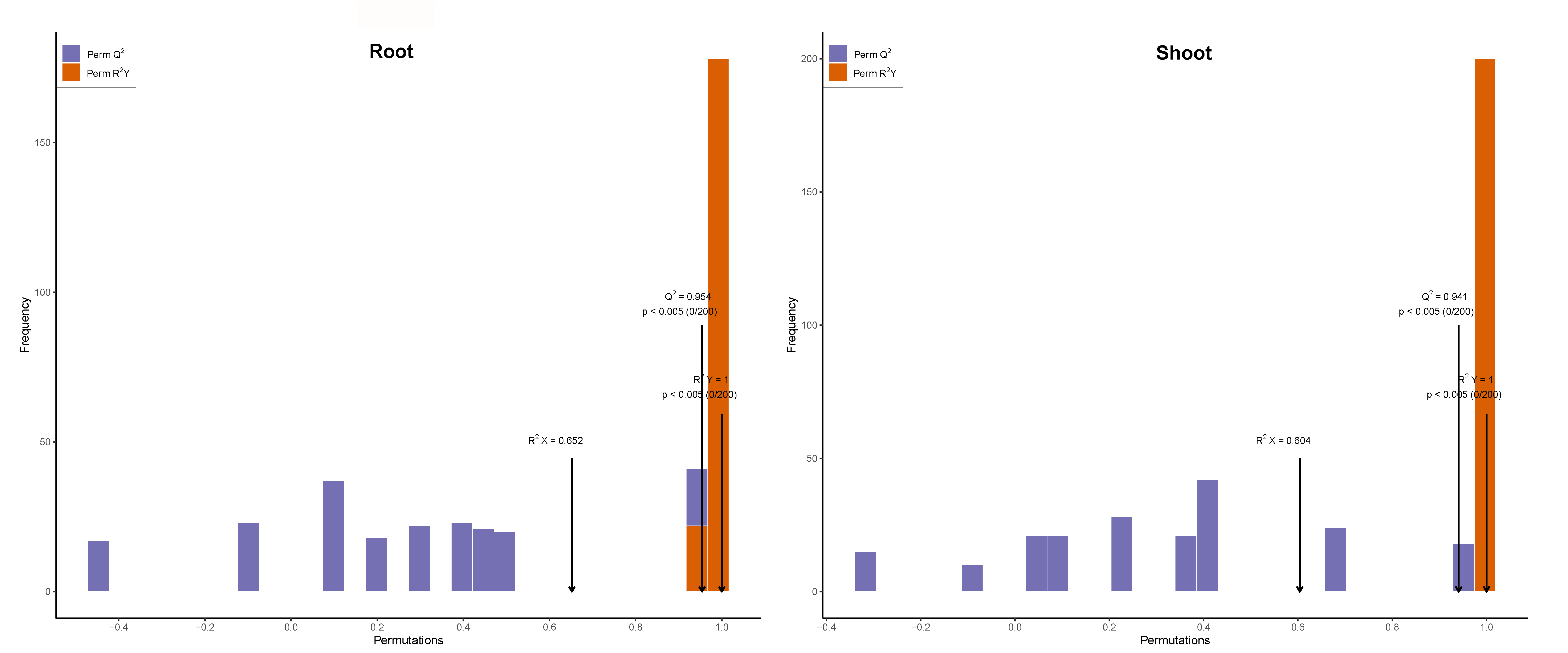

Supplement: Supplementary file 1 [file Image1.tif]
