# Supplementary material for: The responses of rice plant to tricyclazole at the transcriptome and metabolome levels
Source: Front Plant Sci. 2026 Feb 3;17:1723722. doi: 10.3389/fpls.2026.1723722 (PMC12909226; doi:10.3389/fpls.2026.1723722)
Supplement: Supplementary file 2 [file Table1.docx]

Figure S1. Verification of OPLS-DA model. Orange represents the R^2^Y model, purple represents the Q^2^ model, and black arrows represent the R^2^X, R^2^Y, and Q^2^ values.
