# Supplementary material for: The responses of rice plant to tricyclazole at the transcriptome and metabolome levels
Source: Front Plant Sci. 2026 Feb 3;17:1723722. doi: 10.3389/fpls.2026.1723722 (PMC12909226; doi:10.3389/fpls.2026.1723722)
Supplement: Supplementary file 3 [file Table2.docx]

Supplementary tables

### Table S1 RNA sample assessment

| **Sample** | Concentration (ng/μL) | Volume (μL) | Total amount (μg) | RIN |
| --- | --- | --- | --- | --- |
| CK-Root-1 | 211 | 45 | 9.5 | 8.2 |
| CK-Root-2 | 248 | 45 | 11.16 | 8.4 |
| CK-Root-3 | 128 | 45 | 5.76 | 8.2 |
| CK-Shoot-1 | 159 | 45 | 7.16 | 8.6 |
| CK-Shoot-2 | 263 | 45 | 11.84 | 8.4 |
| CK-Shoot-3 | 347 | 45 | 15.62 | 8.3 |
| P-Root-1 | 291 | 45 | 13.1 | 8.1 |
| P-Root-2 | 214 | 45 | 9.63 | 8.1 |
| P-Root-3 | 240 | 45 | 10.8 | 8.0 |
| P-Shoot-1 | 247 | 45 | 11.12 | 8.4 |
| P-Shoot-2 | 259 | 45 | 11.66 | 8.4 |
| P-Shoot-3 | 357 | 45 | 16.07 | 8.4 |

### Table S2 Summary of sequencing output statistics

| **Sample** | **Raw Reads** | **Clean Reads** | **Reads mapped** | **Unique mapped** | **Q20 (%)** | **Q30 (%)** | **GC Content (%)** |
| --- | --- | --- | --- | --- | --- | --- | --- |
| CK-Root-1 | 65132012 | 62107338 | 47505072 (76.49%) | 45826844 (73.79%) | 99.34 | 97.65 | 47.58 |
| CK-Root-2 | 70580868 | 67992244 | 48471833 (71.29%) | 46852747 (68.91%) | 99.39 | 97.82 | 47.04 |
| CK-Root-3 | 78156508 | 75848150 | 53956695 (71.14%) | 52053124 (68.63%) | 99.39 | 97.81 | 46.95 |
| CK-Shoot-1 | 64679994 | 62699174 | 61254774 (97.70%) | 58743049 (93.69%) | 99.34 | 97.65 | 50.33 |
| CK-Shoot-2 | 63469030 | 61506986 | 60028744 (97.60%) | 57481087 (93.45%) | 99.29 | 97.5 | 50.27 |
| CK-Shoot-3 | 56109402 | 54491200 | 53272300 (97.76%) | 51006139 (93.60%) | 99.28 | 97.47 | 50.94 |
| P-Root-1 | 61464776 | 59188132 | 50290906 (84.97%) | 48414394 (81.80%) | 99.34 | 97.67 | 48.18 |
| P-Root-2 | 63772904 | 61826460 | 52020805 (84.14%) | 50078903 (81.00%) | 99.33 | 97.62 | 48.44 |
| P-Root-3 | 71848750 | 70061614 | 60546503 (86.42%) | 58296439 (83.21%) | 99.29 | 97.48 | 48.53 |
| P-Shoot-1 | 56958318 | 55235984 | 54113524 (97.97%) | 51712642 (93.62%) | 99.3 | 97.51 | 49.79 |
| P-Shoot-2 | 58457850 | 56477252 | 55274040 (97.87%) | 52956891 (93.77%) | 99.33 | 97.6 | 49.73 |
| P-Shoot-3 | 66067692 | 63065458 | 61593676 (97.67%) | 58906992 (93.41%) | 99.35 | 97.71 | 49.9 |
